# Associations between digital media use and lack of physical exercise among middle-school adolescents in Korea

**DOI:** 10.4178/epih.e2023012

**Published:** 2023-01-10

**Authors:** Gyeongmin Kim, Hyunsuk Jeong, Hyeon Woo Yim

**Affiliations:** Department of Preventive Medicine, College of Medicine, The Catholic University of Korea, Seoul, Korea

**Keywords:** Adolescent, Sex characteristics, Video games, Screen time, Exercise

## Abstract

**OBJECTIVES:**

The reported effects of digital media overuse on physical activity among adolescents are inconsistent. This study examined the association between hours of digital media use and lack of moderate-intensity physical exercise (mPE) according to the type of digital media.

**METHODS:**

This study included 1,837 middle school students from the iCURE (Internet user Cohort for Unbiased Recognition of gaming disorder in Early Adolescence) study conducted in Korea. Hours spent using digital media were measured by self-reported daily usage time for Internet games, messengers, social media, and watching game streaming on weekdays. Lack of mPE was defined as performing a minimum of 30 minutes at a time less than twice weekly. Multivariable logistic regression analysis stratified by sex was performed.

**RESULTS:**

Among male students, the group with the highest hours of using either Internet games or watching game streaming was more likely to lack mPE than each non-user group. In contrast, among male students, the group using either messengers or social media had a higher rate of mPE compared to each non-user group. Female students showed no association between hours spent using Internet games, messengers, social media, or watching game streaming and a lack of mPE.

**CONCLUSIONS:**

Among male middle school students in Korea, the excessive use of Internet games or watching game streaming was associated with a lack of mPE. Thus, guidelines should be established regarding adolescent use of internet games and watching game streaming.

## INTRODUCTION

Although participation in appropriate physical activities during adolescence has benefits such as improved sleep quality, enhanced brain executive function, reduced risk and severity of depression, reduced anxiety symptoms and its severity, improved quality of life, and enhanced physical function [1], worldwide, four out of five adolescents aged between 11 and 17 lack sufficient physical activity [2]. Sedentary behaviors that consume little energy, especially screen-based activities, are potential risk factors for adolescent health and well-being [3].

Some studies have reported about decreased physical activity in adolescents with increasing screen time [4-6]. However, another study reported that physical and screen-based activities, such as using a smartphone while running on a treadmill, did not displace each other but rather could be compatible or independent [7].

Previous studies focused primarily on TV watching and computer use as indicators of traditional screen time and investigated their effects on adolescent physical activity. Most studies also focused on devices such as TVs, PCs, video game consoles, and mobile phones, or total screen time combining all these devices as an exposure variable [6,8-10]. As the way of interacting with the screen has changed rapidly over the past decade, a more sophisticated approach and understanding of the impact of new digital media is required [3]. Media can now be accessed at any time regardless of the type of device and the place of use; thus, adolescents’ physical activities may be influenced by the type, nature, and usage pattern of media content rather than the screen time of using media devices. In addition, since the types and patterns of the predominantly used media differ by sex [11], an analysis of the relationship between excessive use of digital media and lack of exercise according to digital media types stratified by sex is required.

The present study divided digital media into types including Internet game use, game streaming watching, messenger use, and social media use, and determined whether excessive use of each media was associated with a lack of exercise in adolescents.

## MATERIALS AND METHODS

### Study subjects

**Figure 1.**
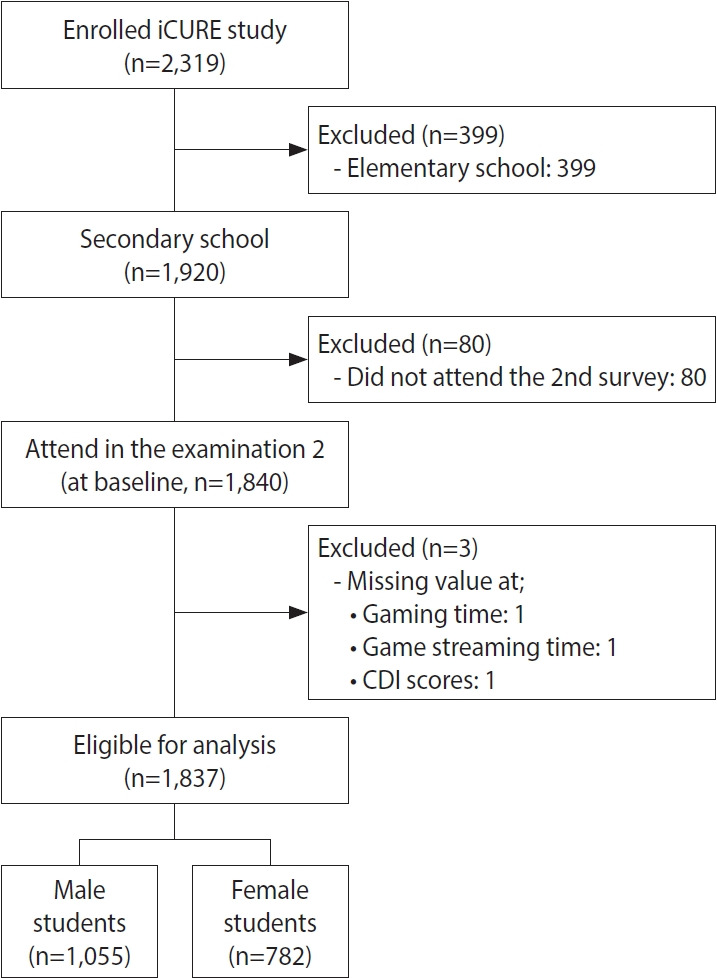
Flow diagram of subjects selection. iCURE, Internet user Cohort for Unbiased Recognition of gaming disor der in Early Adolescence; CDI, Children’s Depression Inventory.

The iCURE (Internet user Cohort for Unbiased Recognition of gaming disorder in Early Adolescence) study started with a baseline assessment in 2015, and three follow-up surveys were conducted yearly until 2018. A total 2,319 3rd, 4th, and 7th graders enrolled form Seoul and Uijeongbu city in Gyeonggi-do. The study protocol has been described in detail elsewhere [12]. Because elementary and middle school students in Korea have different average daily media usage times and moderate-intensity physical exercise (mPE) rates, the present study included only middle school students (7th graders) to ensure the homogeneity of the study population. Of the 1,920 middle school students registered in the cohort, 80 students who did not participate in the first-year followup due to transfer or study abroad were excluded. Of the 1,840 remaining students, two were excluded due to missing media usage time, and one was excluded due to missing variables on depressive symptoms among potential confounding variables. Therefore, the final analysis included a total of 1,837 students (Figure 1).

### Types of digital media and usage time

Digital media types were classified into Internet games, messengers, social media, and watching game streaming. As examples of each type included in the survey questionnaire, the Internet games included ‘League of Legends’, ‘FIFA Online’, ‘Minecraft’, ‘Overwatch’, and ‘Maple Story’; the messengers included Kakao Talk, Dontok, and Line; the social media included Facebook, Instagram, Twitter, Kakao Story, and blogs; and the game streaming included Afreeca TV and YouTube [13].

Since middle school students’ digital media usage time may be much lower than usual during exam periods, the ‘past 3 months’ rather than ‘past 1 month’ was used as the reference period to investigate digital media usage time. The digital media usage time during weekdays was set as an exposure variable due to the significant difference in digital media usage times between weekdays and weekends.

### Lack of moderate-intensity physical exercise

mPE was measured using a dichotomous question answering yes or no regarding regularly performing exercises including ‘tennis/badminton/squash,’ ‘soccer/foot volleyball/basketball/skiing’, or ‘other physical activities’ (> 30 minutes at a time, at least 2-3 times/wk). Participants responding ‘yes’ to ‘other physical activities,’ were asked to write the name of the activities. The categorization of the ‘other physical activities’ as mPE was determined based on adolescents’ level of physical activity in the ‘Physical Activity Guidelines for Koreans’ [14]. If the participants responded ‘no’ to all three questions above, or if the activities described in the other physical exercises did not reach the level of moderate intensity, they were categorized as ‘lack of mPE’.

### Confounding variables

Self-reported depressive symptoms were assessed using the Korean version of the Children’s Depression Inventory (CDI) [15], which was measured with a score of 0-2 points over the past 2 weeks, ranging from 0 points to 54 points, which is the sum of the scores of 27 questions. The higher the score, the higher the level of depressive symptoms. This study defined a score of 22 or more as having depressive symptoms [15]. The Cronbach’s alpha was 0.90.

State anxiety levels were assessed using the Korean State Anxiety Inventory for Children (SAIC) [16]. State anxiety refers to a state of subjective and conscious anxiety that varies in intensity and changes over time as a temporary emotional state of humans. The SAIC was measured, and the total score ranged from 20 points to 60 points, which is the sum of the scores of a total of 20 items. The higher the score, the higher the level of state anxiety. This study defined a score of 40 or more as having state anxiety symptoms [16]. The Cronbach’s alpha was 0.93.

Aggression level was measured using the Korean version of the Aggression Questionnaire (AQ-K) [17]. The AQ-K contains 29 items on a 5-point Likert scale, ranging from 1 point for ‘strongly disagree’ to 5 points for ‘strongly agree.’ The higher the total score, the higher the level of aggression. The Cronbach’s alpha in this study was 0.86.

The maternal educational level was obtained from the self-reported data from the participants’ guardians. It was classified as ‘below college graduate,’ ‘college graduate and higher, and ‘unknown’ when not answered.

The maternal educational level was obtained from the self-reported data from the participants’ guardians. It was classified as ‘below college graduate,’ ‘college graduate and higher, and ‘unknown’ when not answered.Regarding private tutoring hours, the total hours at private educational institutes or extracurricular hours were reported in hours and minutes. The analysis converted it into the average time spent on private tutoring per day during weekdays in minutes. Based on the median times, the private tutoring times were classified as high or low.

### Statistical analysis

SAS version 9.4 (SAS Institute Inc., Cary, NC, USA) was used to calculate the descriptive statistics and perform a multivariable analysis. Because the types of media content and usage time that are mainly used differ by sex in adolescents [18], the analysis was stratified into male and female. Time spent on digital media was divided into non-user and user groups. The user group was further divided into four groups based on quartiles for each type of digital media.

The differences in mPE rates according to the usage time compared to the non-user group stratified by the type of digital media were examined by chi-square tests. Bonferroni correction for the p-value was not performed and exploratory interpretation was performed. A trend analysis was performed to assess the potential trend in moderate-intensity exercise rate between the lowest quartile group with the lowest usage time (first quartile group) and the highest quartile group with the higher usage time (fourth quartile group).

To determine whether the usage time for each type of digital media was independently associated with the lack of exercise, a multivariable logistic regression analysis was performed after adjusting potential confounders and estimated the adjusted odds ratio (aOR) and 95% confidence interval (CI).

Owing to the possibility of false reporting in self-reported surveys on Internet game use in adolescents [19], a sensitivity analysis was performed to identify any difference in mPE rate according to the time of Internet game use after excluding 116 subjects with inconsistencies in questionnaire responses related to game use.

### Ethics statement

This secondary analysis of cohort data was approved by the Institutional Review Board at the Catholic University of Korea, Songeui Campus (MC21EASI0048). Anonymized data were used for the analysis.

## RESULTS

Of the 1,873, 1,055 (57.5%) and 782 (42.6%) were male and female students, respectively (Table 1). Among these, 4.4% of male subjects had depressive symptoms, and 7.3% had state anxiety symptoms, which were lower than the rates of 7.7% and 13.2% in the female students. The aggression level of the male students was slightly higher than that of female students (p< 0.001). The weekday private tutoring time and maternal educational level did not differ significantly between male and female students.

The amount of time spent on each type of digital media differed by sex. The median usage times spent playing Internet games and watching game streaming were 70 minutes and 30 minutes, respectively, for male students, which were higher than those of female students (p< 0.001). In contrast, the median times that female students spent on messengers and social media were 60 minutes, respectively, twice as high as that in male students (p< 0.001).

The mPE rates were 59.1% and 26.3% in male and female, respectively.

**Figure 2.**
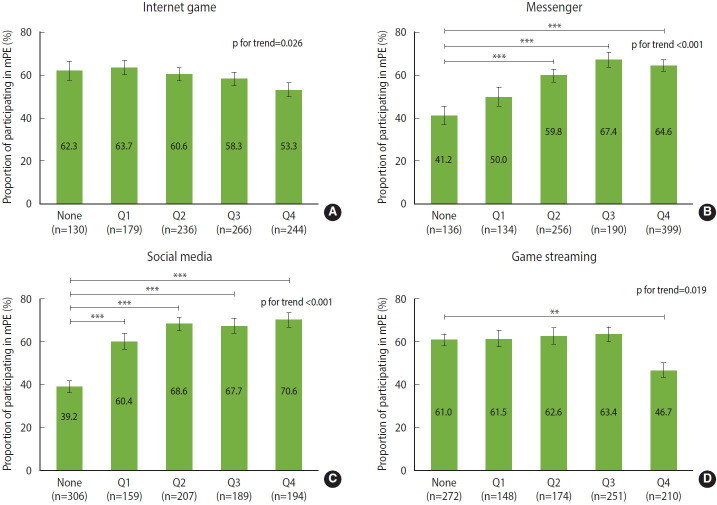
Proportion of participating in mPE in digital media usage time among male students. (A) Internet game, (B) messenger, (C) social media, and (D) game streaming. mPE, moderate-intensity physical ex ercise; None, not use the media; Q1, 1st quartile (the lowest 25%); Q2, 2nd quartile; Q3, 3rd quartile; Q4, 4th quartile (the highest 25%). p-value were obtained using the χ^2^ test and the Cochran-Armitage test.

Male students showed no significant difference in mPE rates between the Internet game non-user and each quartile user groups, however Internet game usage time increased, the mPE rate tended to decrease (p for trend= 0.026) (Figure 2A, Supplementary Material 1).

Regarding messenger use among digital media, the mPE rate was significantly higher in the second, third, and fourth quartiles groups of messenger usage time compared to the non-user group (2nd quartile: p= 0.001; 3rd and 4th quartile: p< 0.001), also messenger usage time increased, the mPE rate tended to increase (p for trend < 0.001) (Figure 2B, Supplementary Material 2).

Regarding social media among digital media, the mPE rate was significantly higher in the 1st, 2nd, 3rd, and 4th quartiles groups of social media usage time compared to the non-user group (1st, 2nd, 3rd, and 4th quartiles, p< 0.001), also time spent on social media increased, the mPE rate tended to increase (p for trend < 0.001) (Figure 2C, Supplementary Material 3).

Regarding the watching of game-streaming, mPE rate was significantly lower in the 4th quartile group with the highest game-streaming watching time compared to the non-user group (p= 0.002) (Figure 2D). A trend was observed between the time spent watching game streaming and mPE (p for trend= 0.019) (Figure 2D, Supplementary Material 4).

**Figure 3.**
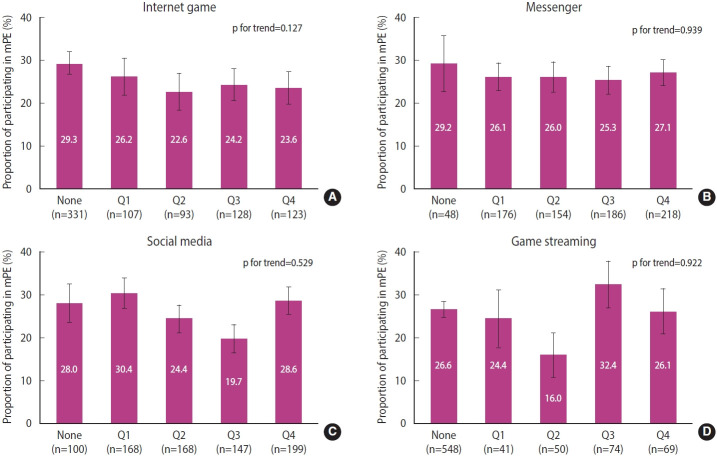
Proportion of participating in mPE in digital media usage time among female students. (A) Internet game, (B) messenger, (C) social media, and (D) game streaming. mPE, moderate-intensity physical ex ercise; None, not use the media; Q1, 1st quartile (the lowest 25%); Q2, 2nd quartile; Q3, 3rd quartile; Q4, 4th quartile (the highest 25%). p-value were obtained using the χ^2^ test and the Cochran-Armitage test.

The female students showed no significant difference in mPE rate between media user and non-user groups in all four types of digital media. (Figure 3, Supplementary Materials 1-4).

**Figure 4.**
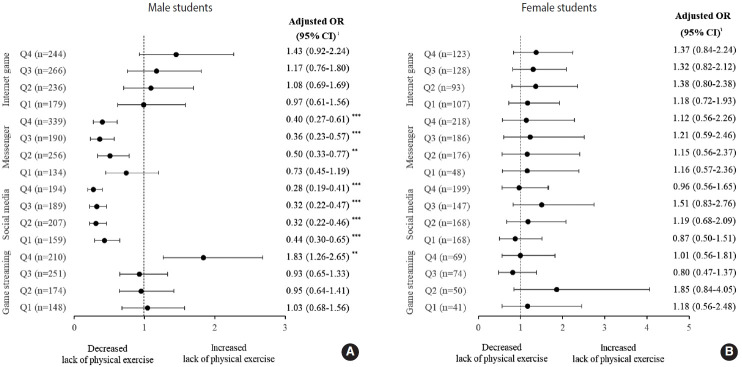
Multivariable logistic regression analysis of association between digital media use and lack of moderate intensity physical exercise. (A) male students and (B) female students. OR, odds ratio; CI, confidence interval; Q1, 1st quartile (the lowest 25%); Q2, 2nd quartile; Q3, 3rd quartile; Q4, 4th quartile (the highest 25%). ^1^Adjusted for maternal educational level, aggression, children’s depression, state anxiety, and time spent on private tutoring. **p<0.01, ***p<0.001.

aORs were calculated after adjusting potential confounders with the non-user group as a reference. Male students showed no relationship between time spent on Internet games and the mPE rate (Figure 4, Supplementary Material 1). However, the 4th quartile group of game streaming watching had a significantly higher mPE rate (aOR, 1.83; 95% CI. 1.26 to 2.65) (Figure 4, Supplementary Material 4). The 2nd, 3rd, and 4th quartile groups of messenger usage time had a significantly lower mPE rate (2nd quartile: aOR, 0.50; 95% CI, 0.33 to 0.77; 3rd quartile: aOR. 0.36; 95% CI, 0.23 to 0.57; 4th quartile: aOR, 0.40; 95% CI, 0.27 to 0.61) (Figure 4, Supplementary Material 2). In addition, the mPE rate was significantly lower in all social media user groups (1st quartile: aOR, 0.44; 95% CI, 0.30 to 0.65; 2nd quartile: aOR, 0.32; 95% CI, 0.22 to 0.46; 3rd quartile: aOR, 0.32; 95% CI, 0.22 to 0.47; 4th quartile: aOR, 0.28; 95% CI, 0.19 to 0.41) (Figure 4, Supplementary Material 3).

Female students showed no relationship between time spent on any digital media type and the mPE rate (Figure 4, Supplementary Materials 1-4).

**Figure 5.**
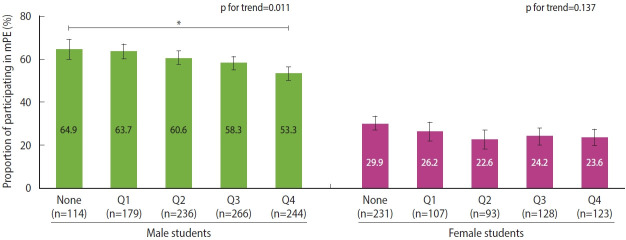
Sensitivity analysis (excluding participants who had discrepancy on whether or not to use games). Proportion of participating in moderate-intensity physical exercise (mPE) in digital media usage time. None, not use the media; Q1, 1st quartile (the lowest 25%); Q2, 2nd quartile; Q3, 3rd quartile; Q4, 4th quartile (the highest 25%). *p<0.05.

After excluding 116 subjects with suspected false reports of time spent on Internet games, the sensitivity analysis showed that male students had a significantly lower moderate-intensity exercise rate in the 4th quartile group with the highest Internet game usage time compared to the non-user group (p= 0.038), suggesting a negative tendency between the time spent on Internet game use and the mPE rate (p= 0.011) (Figure 5). In the multivariable logistic regression analysis, the mPE rate was significantly higher in male students the 4th quartile of Internet game usage time compared to the non-user group (aOR, 1.61; 95% CI, 1.01 to 2.59) (Supplementary Material 5).

## DISCUSSION

This study examined the potential difference in mPE rates according to the time spent on each type of digital media among adolescents. Male students showed no significant difference in mPE rates according to the time spent on Internet games. However, the mPE rate was significantly decreased in the group that excessively watched game streaming compared to the non-user group. In contrast, the mPE rate increased significantly in the second, third, and fourth quartile groups of messenger use compared to the non-user group, and the mPE rate increased significantly in all quartile groups of social media use compared to the non-user group.

The World Health Organization (WHO) guidelines for adolescent physical activity recommend moderate physical activity for an average of 60 minutes or more daily or vigorous aerobic or muscle exercise at least 3 days a week [20]. The physical activity rate of Korean adolescents meeting this standard was 5.8% [2]. The outcome variable in this study was regular mPE for a minimum of 30 minutes at least twice weekly, an operational definition that differs from the WHO guidelines. The 2016 National Sports Survey data [21], which was performed at the same time as the present study and used nearly the same definition as the outcome variable, reported a participation rate in regular physical activity of middle school students of 44.7%, which was similar to the moderate-intensity exercise rate of 45.1% in the present study.

Among the types of digital media, the relationships between mPE and time spent on Internet games was expected to be similar to that of time spent watching the game initially. However, the results of this study showed different results, possibly due to differences in sensitivity in exposure evaluation according to the type of digital media.

Adolescents’ under-report socially undesirable risk behaviors, such as game use, in self-reported measures [19]. In particular, lying about time spent on game time is a diagnostic criterion for Internet gaming disorder. Moreover, a tendency to overestimate time spent on social media in self-reports has been reported [22]. However, false reports or related tendencies regarding watching game streaming have not been examined. Unlike the excessive use of Internet games, the excessive watching of game videos is not yet viewed negatively in Korean society; hence, the false report rate was expected to be low when measuring time spent on game streaming.

The analysis of the relationship distortion caused by false reports in excessive Internet game users included 1,721 subjects, excluding 116 subjects with discrepancies in Internet game questions suggesting false reports. The mPE rate was significantly decreased in excessive users of Internet games, similar to those who excessively watched game streaming.

A cross-sectional study in Norway that examined the relationships between total screen time per day, including TV, PC and games, and moderate-intensity physical activities measured by accelerometer from self-reported nationally representative data of 3,920 children and adolescents aged 9 years and 15 years old reported a negative relationship in which physical activity decreased by an average of 2 min/day as total screen time increased by 1 hr/day [23]. The results of the sensitivity analysis in the present study confirmed the negative relationship between time spent on Internet games and the mPE rate. The mPE significantly decreased in excessive users compared to non-users.

On the contrary, as the use of messenger and social media increased among male students, the rate of mPE also increased. A cross-sectional study of 187 middle school and university students in Hong Kong that investigated the relationship between exposure time of each media on smartphones and time for moderate-intensity physical activities measured with an accelerometer reported that increased time spent using messengers and social media was related to increased moderate-intensity physical activity [7], a finding consistent with the results of the present study. A qualitative study of adolescents using social media in the United Kingdom reported that male students’ motivation for using social media was to communicate with friends and access health information related to their appearance [24]. Since people with many physical activities can use messengers and social media to share photos and health information [7], a positive relationship may exist between social media use and moderate-intensity physical activity.

Female students showed no significant relationship between mPE rate and social media use and time spent performing them, including Internet games, messengers, social media, and watching game streaming. In a study that analyzed 19,543 middle and high school students in the United States, physical activity increased as the frequency of using social media increased in physically active students. Physical activity decreased as the frequency of using social media increased among students with little physical activity, suggesting a difference depending on the level of physical activity [25]. A study in Norway that analyzed data from 200,615 adolescents aged 11-15 years in 39 North American and European countries reported decreased moderate-intensity physical activity in both males and females when the total time spent on TV, PC, and gaming exceeded 2 hours [26].

Only 2.8% of female Korean students met the WHO physical activity guideline level, a rate significantly lower than the 19.5% of female students reported in the United States, a representative North American country, and 8.5% of Italian female students, a representative Southern European country [2]. Therefore, no relationship between the mPE rate according to the type of digital media was observed because of the overall lack of physical activity in adolescent Korean female students.

The rate of lack of mPE was 35.1% in male students who did not use Internet games. Thus, the odds ratio (OR), the size of the association derived from the study, may overestimate the relative risk (RR) [27]. When the OR was 1.62, the converted RR was 1.33. Assuming that excessive use of Internet games in male middle school students was the direct cause of lack of mPE, this finding suggested that excessive use of Internet games increased the rate of lack of exercise by 1.33-fold.

The strength of this study is that instead of total screen time, which does not differentiate media types, the usage times of examples of each type of digital media preferred by the research subjects were assessed to identify the relationships between moderate-intensity exercise rates for each type of digital media. Second, previous studies used ambiguous and broad definitions of physical activity. However, this study was limited to mPE to identify its relationship with time spent on digital media among middle school students.

The limitations of this study were as follows: first, as a cross-sectional study, only relationships and not causal associations could be identified. Second, since time spent on digital media and participation in moderate-intensity exercise were subjectively measured, the students may have inflated their exercise participation due to their desire for social recognition or underreported their media usage time due to a bias toward social desirability. Accordingly, a sensitivity analysis was performed to exclude false reports of Internet game usage time, which identified the relationship. Third, the determination of the independent effect of the type of media, which is an exposure variable, did not adjust for the time spent using other media types. Since two or more media types, such as Internet games, watching game streaming, and messengers and social media, are linked as one act, they were practically difficult to separate. Therefore, we tried calculating the observed media use as an odds ratio. Fourth, since digital media use patterns and moderate-intensity physical activity can be affected by country, region, and individual characteristics, and this study targeted students attending schools in metropolitan areas in Korea, care should be taken when generalizing the results in other countries, rural areas, and out-of-school youth. Fifth, the association between social media use and physical activity of less than moderate intensity in female students could not be analyzed. Because female students are highly interested in social media and their excessive usage, the association with lower-intensity physical activity requires investigation. The present study could not examine this association because these data were not assessed. Future studies are needed to study the relationship between social media use according to sex and physical activity level.

When male middle school students excessively use online games and watch game streaming, the moderate-intensity exercise rate may decrease. Future research on this relationship and the establishment of guidelines for digital media use are needed.

## Figures and Tables

**Table 1.** Characteristics stratified by sex among 1,837 iCURE study participants

Characteristics	Male students	Female students	p-value^1^
Demographic factors	1,055 (57.5)	782 (42.6)	
Age (yr)	14 (14, 15)	14 (14, 15)	0.203
Maternal educational level			
<College	337 (31.9)	274 (35.0)	0.139
≥College	700 (66.4)	488 (62.4)	
Unknown	18 (1.7)	20 (2.6)	
Average time spent on private tutoring (min/weekday)	96 (36-174)	108 (38-174)	0.283
Psychological factors			
Depression symptoms^2^	46 (4.4)	60 (7.7)	0.003
State anxiety symptoms^3^	77 (7.3)	103 (13.2)	<0.001
Aggression^4^	54 (47-65)	52 (46-62)	<0.001
Average time spent on media (min/weekday)			
Internet game	70 (30-120)	10 (0-60)	<0.001
Messenger	30 (5-60)	60 (20-120)	<0.001
Social media	30 (0-60)	60 (25-180)	<0.001
Watching game streaming	30 (0-80)	0 (0-20)	<0.001
Participation in moderate intensity physical exercise^5^	623 (59.1)	206 (26.3)	<0.001

Values are presented as number (%) or median (interquartile range). iCURE, Internet user Cohort for Unbiased Recognition of gaming disorder in Early Adolescence.

1 Using the chi-squared test and the Wilcoxon rank sum test.

2 Children’s Depression Inventory scores ≥22.

3 State Anxiety Inventory for Children scores ≥40.

4 Aggression Questionnaire range: 29-145.

5 ≥Participating in moderate intensity physical exercise on 2 days of the week (more than 30 minutes at a time).

## SUPPLEMENTARY MATERIALS

Korean version is available at https://www.e-epih.org/.

Supplementary Material 1. Associations between time spent on playing Internet game and lack of moderate intensity physical exercise (n=1,837)

Supplementary Material 2 Associations between time spent on Messenger and lack of moderate intensity physical exercise (n=1,837)

Supplementary Material 3 Associations between time spent on Social media and lack of moderate intensity physical exercise (n=1,837)

Supplementary Material 4 Associations between time spent watching Game streaming and lack of moderate intensity physical exercise (n=1,837)

Supplementary Material 5 Sensitivity analysis of associations between time spent on playing Internet game and lack of moderate intensity physical exercise (n=1,721)
