# Supplementary material for: Associations between digital media use and lack of physical exercise among middle-school adolescents in Korea
Source: Epidemiol Health. 2023 Jan 10;45:e2023012. doi: 10.4178/epih.e2023012 (PMC10581895; doi:10.4178/epih.e2023012)
Supplement: Supplementary file 1 [file epih-45-e2023012-Korean-Supplementary.docx]

**Associations between digital media use and lack of physical exercise among middle school adolescents in Korea**

**한국 중학생에서 디지털 미디어 유형별 사용시간과 중강도 신체운동 부재와의 연관성**

김경민^1^, 정현숙^1^, 임현우^1^

^1^ 가톨릭대학교 의과대학 예방의학교실

**소제목 : 청소년에서의 디지털 미디어 사용과 신체운동**

**CORRESPONDENCE**

Name : Hyeon Woo Yim

Address : Department of Preventive Medicine, College of Medicine, The Catholic University of Korea, 222 Banpo-daero, Seocho-gu, Seoul 06591, Korea

Tel : +82 2 3147 8376

Fax : +82 2 532 3820

E-mail : y1693@catholic.ac.kr

**CONFLICT OF INTEREST**

The authors have no conflicts of interest to declare for this study

**FUNDING**

None

**초록**

**목적**: 청소년들에서 미디어 과다 사용이 신체활동에 미치는 영향은 일관적이지 않았다. 디지털 미디어 유형으로 구분하여 디지털 미디어 사용시간에 따른 중강도 신체운동 부재와의 연관성을 확인하고자 하였다.

**방법**: 한국에서 수행된 iCURE 연구에서 중학생 1,837명의 자료를 이용하였다. 디지털 미디어 사용시간은 자가 보고된 인터넷게임, 메신저, 소셜미디어 사용과 게임 스트리밍 시청에 대한 평일 일일 사용시간을 이용하였다. 한번에 30분 이상의 중강도 수준 이상 운동을 주 2회 미만 실시하는 경우 중강도 신체운동 부재로 정의하였다. 성별로 층화하여 다중 로지스틱 회귀 분석을 수행하였다.

**결과**: 남학생에서 인터넷게임 사용 및 게임 스트리밍 시청 시간이 가장 높은 집단은 비사용군에 비해 중강도 신체운동 부재율이 높았다. 반면 남학생에서 메신저 및 소셜미디어 사용군은 비사용군에 비해 중강도 신체운동률이 높았다. 여학생에서 인터넷게임, 메신저, 소셜미디어의 사용시간과 게임 스트리밍 시청시간은 중강도 신체운동 부재와 연관성이 없었다.

**결론**: 한국의 남자 중학생에서 인터넷게임 과다사용 및 게임 스트리밍 과다 시청은 중강도 신체운동 부재율과 연관성이 있었다. 청소년을 대상으로 인터넷게임 사용 및 게임 스트리밍 시청에 대한 가이드라인 제정과 적용이 필요하다.

**주제어**: 청소년, 성별, 인터넷게임, 게임 스트리밍, 스크린 타임, 신체운동

**Abstract**

**Objectives:** The reported effects of digital media overuse on physical activity among adolescents are inconsistent. This study examined the association between hours of digital media use and lack of moderate-intensity physical exercise according to the type of digital media.

**Method:** This study included 1,837 middle school students from the iCURE study conducted in Korea. Hours spent using digital media were measured by self-reported daily usage time for internet games, messengers, social media, and watching game streaming on weekdays. Lack of moderate-intensity physical exercise was defined as performing a minimum of 30 minutes at a time less than twice weekly. Multivariate logistic regression analysis stratified by gender was performed.

**Result:** Among male students, the group with the highest hours of using either internet games or watching game streaming was more likely to lack moderate-intensity physical exercise than each non-user group. In contrast, among male students, the group using either messengers or social media had a higher rate of moderate-intensity physical exercise compared to each non-user group. Female students showed no association between hours spent using Internet games, messengers, social media, or watching game streaming and a lack of moderate-intensity physical exercise.

**Conclusion:** Among male middle school students in Korea, the excessive use of Internet games or watching game streaming was associated with a lack of moderate-intensity physical exercise. Thus, guidelines should be established regarding adolescent use of internet games and watching game streaming.

**Keywords**: Adolescents, gender, Internet games, game streaming, screen time, physical exercise

**서론**

청소년기의 적절한 신체활동 참여는 수면의 질 개선, 뇌의 실행 기능 증진, 우울증 위험 및 심각도 감소, 불안 증상 및 심각도 감소, 삶의 질 개선, 신체적 기능 증진의 이점이 있음에도 [1], 전세계 11-17세 청소년 5명 중 4명은 신체활동이 불충분한 것으로 보고되었다 [2]. 에너지를 거의 소비하지 않고 앉아 있는 좌식 행동, 그 중에서도 특히 스크린 기반 활동은 청소년의 건강과 웰빙에 대한 잠재적인 위험 요소로 밝혀졌다 [3].

청소년에서 스크린 시간이 증가함에 따라 신체활동 감소를 보고한 연구들이 있다 [4-6]. 반면 런닝머신을 하며 스마트폰을 사용하는 것처럼 신체활동과 스크린 기반 활동은 대체되는 것이 아닌 서로 양립하거나 독립적일 수 있는 관계로 보고한 연구도 있었다[7].

이전 연구들은 전통적인 스크린타임의 지표로서 TV시청, 컴퓨터 사용에 주로 초점을 맞추고 청소년들의 신체활동에 미치는 영향을 조사해 왔다. 주로 TV와 PC, 비디오 게임 콘솔, 휴대폰 등의 기기별 스크린 시간에 대한 기기 중심 연구 또는 이들을 합산 총 스크린 시간을 노출변수로 설정한 연구들이 많았다 [6, 8-10]. 지난 10년간 스크린과 상호작용하는 방식이 급격히 변화하면서 새로운 디지털미디어의 영향에 대한 보다 정교한 접근 방식과 이해가 요구되는 시점이다 [3]. 특히 미디어 사용 기기 종류와 사용 장소에 구애받지 않고 같은 미디어 유형에 언제든 접속하여 사용하는 것이 가능하여, 미디어 사용 기기에 대한 스크린 시간보다 주로 사용하는 미디어 콘텐츠의 종류와 성격, 사용패턴에 따라 청소년의 신체활동과의 연관성이 달라질 수 있다. 또한 성별에 따라 주로 사용하는 미디어 유형과 패턴이 매우 다르기 때문에 [11], 성별로 층화하여 디지털 미디어 유형에 따른 디지털미디어 과다사용과 운동 부재와의 연관성을 살펴볼 필요가 있다.

본 연구의 목적은 청소년에서 디지털 미디어 유형을 인터넷게임 사용, 게임 스트리밍 시청, 메신저 사용, 소셜미디어 사용으로 나누어 각 미디어 유형의 과다사용이 운동 부재와 연관성이 있는지를 확인하고자 하였다.

**방법**

**연구대상자**

iCURE(Internet user Cohort for Unbiased Recognition of gaming disorder in Early adolescence)연구는 2015년 기저시점 평가를 시작으로, 2018년까지 1년 단위로 세 번의 추적조사가 실시되었다. 서울과 경기도 의정부에서 초등학교 3학년·4학년, 중학교 1학년 총 2,319명이 등록되었다. 연구 프로토콜은 다른 논문에 자세히 기술되어 있다 [12].

한국의 초등학생과 중학생은 일일 평균 미디어 사용시간이 서로 다르고 중강도 신체운동률도 서로 다르기 때문에 본 연구에서는 연구 대상군의 동질성을 확보하기 위하여 중학생 만을 포함하였다. 코호트에 등록된 중학생 1,920명에서 전출, 유학 등으로 1년차 추적조사에 참여 안한 80명을 제외하였다. 1,840명 중에서 미디어 사용시간이 누락된 2명, 잠재적 혼란변수 중 우울증상이 누락된 1명을 제외하였다. 따라서 최종 분석에서는 총 1,837명을 포함하였다(Fig. 1).

Insert Figure 1 here.

**디지털 미디어 유형과 사용시간**

디지털 미디어 유형은 인터넷게임, 메신저, 소셜미디어, 그리고 게임 스트리밍 시청으로 분류하였다. 각 유형의 예제로서 인터넷게임은 ‘리그 오브 레전드, 피파 온라인, 마인 크래프트, 오버워치, 메이플스토리’, 메신저는 ‘카카오톡, 돈톡, 라인’, 소셜미디어는 ‘페이스북, 인스타그램, 트위터, 카카오스토리, 블로그’, 게임 스트리밍 시청은 ‘아프리카TV, 유튜브’플랫폼을 설문 문항에 제시하여 조사하였다 [13].

중학생들은 시험기간에 의해 디지털 미디어 사용시간이 평소 사용량보다 매우 낮아질 가능성이 있기 때문에 ‘지난 한 달’ 보다는 ‘지난 3개월’을 준거기간으로 하여 디지털 미디어 사용시간을 조사하였다. 주중과 주말의 디지털 미디어 사용시간의 차이가 크기 때문에 주중의 디지털 미디어 사용시간을 노출변수로 설정하였다.

**중강도 신체운동 부재**

중강도 신체운동 여부는 규칙적인(한 번에 30분 이상, 1주일에 적어도 2-3회 이상) 주기로 실시하는 ‘테니스/배드민턴/스쿼시’, ‘축구/족구/농구/스키’, ‘기타 신체활동’에 대해 ‘예/아니오’ 의 두 가지 응답 범주로 확인하였다. ‘기타 신체활동’으로 응답한 경우에는 그 활동명도 함께 기재하도록 하였다. ‘기타 신체활동명’에 기재된 활동이 중강도 신체운동에 해당되는지 여부를‘한국인을 위한 신체활동 지침서’[14]에 제시된 청소년 신체활동 수준을 기준으로 결정하였다. 위의 세 문항에 모두‘아니오’로 대답하거나, 기타 신체운동명에 기재된 활동이 중강도 수준에 미치지 못하는 경우 ‘중강도 신체운동 부재’(이하 ‘운동 부재’)으로 정의하였다.

**혼란변수**

한국어판 아동 우울 척도(Children’s Depression Inventory, CDI) [15]를 사용하여 자가보고에 의한 우울 증상 수준이 평가되었다. 지난 2주 동안 자신의 상태를 문항 당 0-2점의 점수로 측정하며, 총 27개 문항의 점수를 합산한 0-54점 범주 내에서 점수가 높을수록 우울 증상 수준이 높다고 볼 수 있다. 이 연구에서는 22점 이상은 우울 증상이 있음으로 정의하였고 [15], 크론 바흐 알파 값은 0.90이었다.

상태 불안 검사(State Anxiety Inventory for Children, SAIC) [16]를 사용하여 상태 불안 수준이 평가되었다. 상태 불안은 인간의 일시적인 감정의 상태로서 강도가 다양하고 시간의 경과에 따라 변화하는 주관적·의식적 불안상태를 말한다. 총 20개 문항의 점수를 합산한 20-60점 범주를 가진다. 점수가 높을수록 상태 불안 수준이 높다고 볼 수 있다. 이 연구에서는 40점 이상은 상태 불안 증상이 있음으로 정의하였고 [16] 크론 바흐 알파 값은 0.93이었다.

한국판 공격성 척도(Korean Version of Aggression Questionnaire, AQ-K) [17]를 사용하여 공격성 수준이 평가되었다. ‘전혀 그렇지 않다’1점에서 ‘매우 그렇다’5점 리커트 척도로 총 29개 문항이다. 합산된 점수가 높을수록 공격성 수준이 높다고 볼 수 있다. 이 연구에서 크론 바흐 알파 값은 0.86이었다.

인구사회학적 특성 변수 중 하나인 어머니의 교육수준은 보호자의 자가보고 설문자료로부터 얻었으며, 대학교(전문대학) 졸업을 기준으로‘대학교 졸업 미만’와 ‘대학교 졸업 이상’그리고 무응답의 경우 불명(unknown)으로 구분하였다.

사교육 시간은 학원 혹은 과외시간의 평일 총 합산시간을 시간과 분 단위로 응답하도록 하였다. 분석 시에는 분 단위의 평일 하루 평균 사교육 시간으로 변환하여 활용하였다. 중위수를 기준으로 사교육 시간이 많음과 적음으로 구분하였다.

**통계 분석**

SAS 9.4 (SAS Institute Inc., Cary, NC, USA)를 사용하여 연구 변수들의 기술 통계량을산출하고 다변수 분석을 수행하였다.

청소년에서 성별에 따라 주로 사용하는 미디어 컨텐츠 유형과 사용시간이 다르기 때문에 [18] 남녀로 층화하여 분석하였다. 디지털 미디어 사용시간은 비사용군과 사용군으로 분류하였다. 각각의 디지털 미디어 유형의 사용군은 사용시간을 사분위수로 절단하여 4개의 사용시간 군으로 구분하였다.

디지털 미디어 유형별로 비사용자군에 비하여 사용시간에 따라 중강도 신체 운동률(이하 ‘중강도 운동률’)에 차이가 있는지를 카이 제곱 검정으로 확인하였다. 다중 검정에 따른 제1종 오류 상승 방지를 위한, *p*값에 대한 본페로니 교정은 시행하지 않고 탐색적으로 해석하였다. 사용시간이 가장 낮은 최하 사분위군(이하 “1사분위군”)에서 사용시간이 가능 높은 최상 사분위군(이하 “4사분위군”) 간 중강도 운동률에 경향성이 있는지를 확인하기 위하여 경향분석를 수행하였다.

디지털 미디어 유형별 사용시간이 운동 부재와 독립적 연관성이 있는지 알아보기 위해 잠재적 혼란변수를 보정한 다중 로지스틱 회귀분석을 수행하여 보정된 오즈비 (AOR)와 95% 신뢰구간을 추정하였다.

청소년의 인터넷게임 사용 여부 및 인터넷게임 사용장애(IGD) 증상에 대한 연구에서 자가설문에 대한 거짓보고 가능성이 보고되었기 때문에 [19], 게임 사용에 관한 설문 응답이 불일치한 대상자 116명을 제외한 후 인터넷게임 사용시간에 따라 중강도 운동률에 차이가 있는지를 파악하는 민감도 분석을 수행하였다(supplementary table 5).

**윤리적 고려**

코호트 자료에 대한 이 2차 분석은 가톨릭대학교 성의교정 임상연구심의위원회의 심의를 거쳐 승인되었다(MC21EASI0048). 분석에는 익명화된 데이터가 사용되었다.

**결과**

1,873 명의 총 연구대상자 중 1,055명(57.5%)은 남학생이었고, 782명(42.6%)은 여학생이었다(Table 1). 남학생에서 우울 증상이 있는 대상자가 4.4%, 상태 불안 증상이 있는 대상자가 7.3%로 여학생의 7.7%, 13.2% 보다 낮았다. 남학생의 공격성 수준은 여학생의 공격성 수준보다 조금 높았다(*p* < .001). 평일 사교육 시간과 어머니의 교육수준은 남녀 간의 차이가 없었다.

디지털 미디어 유형별 사용시간은 성별에 따라 차이가 있었다. 남학생은 인터넷게임 및 게임 스트리밍의 평균 사용시간 중위수가 각각 70분, 30분으로, 여학생에 비해 높았다(*p* < .001). 반대로 여학생의 메신져 및 소셜미디어 평균 사용시간 중위수는 각각 60분, 60분으로 남학생에 비해 2배 높았다(*p* < .001).

남자 중학생에서의 중강도 운동률은 59.1% 이었고, 여자 중학생에서의 중강도 운동률은 26.3% 이었다.

Insert Table 1 here.

남학생에서 인터넷게임 비사용군과 인터넷게임 사용군들 사이의 중강도 운동률에 유의한 차이가 없었지만(1사분위 *p* = 0.804, 2사분위 *p* = 0.747, 3사분위 *p* = 0.442 4사분위 *p* =0.094), 인터넷게임 사용시간이 증가할수록 전반적으로 중강도 운동률은 감소하는 경향성이 있었다(*p* = 0.026)(Fig. 2A; Supplementary Table 1).

디지털 미디어 유형 중 메신저에서는 메신저 비사용군에 비해 메신저 사용시간 2사분위군, 3사분위군, 4사분위군에서 중강도 운동률이 유의하게 높았으며(2사분위 *p* = 0.001, 3ㆍ4사분위 *p* < .001), 메신저 사용시간이 증가할수록 중강도 운동률이 전반적으로 증가하는 경향성이 발견되었다(*p* < .001)(Fig. 2B; Supplementary Table 2).

디지털 미디어 유형 중 소셜미디어에서는 소셜미디어 비사용군에 비해 소셜미디어 사용시간 1사분위군, 2사분위군, 3사분위군, 4사분위군에서 중강도 운동률이 유의하게 높았으며(1ㆍ2ㆍ3ㆍ4사분위 *p* < .001), 소셜미디어 사용시간이 증가할수록 중강도 운동률도 전반적으로 증가할수록 경향이 나타났다(*p* < .001) (Fig. 2C; Supplementary Table 3).

게임 스트리밍 시청에서는 게임 스트리밍 비시청군에 비해 게임 스트리밍 시청시간이 가장 높은 4사분위군에서 중강도 운동률이 유의하게 낮았으며(*p* = 0.002)(Fig. 2D), 게임 스트리밍 시청 시간과 중강도 운동률 사이의 경향성이 있었다(*p* = 0.019) (Fig. 2D; Supplementary Table 4).

Insert Figure 2 here.

여학생에서는 디지털 미디어 유형 4개 모두에서 미디어 비사용군과 미디어 사용군 사이 중강도 운동률에 유의한 차이가 없었다. (Fig. 3; Supplementary Table 1-4).

Insert Figure 3 here.

aOR은 비사용자군 기준으로 잠재적 혼란요인을 보정한 후 산출되었다. 남학생의 인터넷게임 사용시간과 운동 부재율은 연관성이 없었다(Fig. 4; Supplementary Table 1). 반면게임 스트리밍 시청시간 4사분위군의 운동 부재율은 유의하게 높았다[AOR = 1.83 (95% CI = 1.26 – 2.65)](Fig. 4; Supplementary Table 4).

메신저 사용시간 2사분위군, 3사분위군, 4사분위군은 운동 부재율이 유의하게 낮았다[2사분위 AOR = 0.50 (95% CI = 0.33 – 0.77), 3사분위 AOR = 0.36 (95% CI = 0.23 – 0.57), 4사분위 AOR = 0.40 (95% CI = 0.27 – 0.61)] (Fig. 4; Supplementary Table 2).

또한 모든 소셜미디어 사용군에서 운동 부재율이 유의하게 낮게 나타났다[1사분위 AOR = 0.44 (95% CI = 0.30 – 0.65), 2사분위 AOR = 0.32 (95% CI = 0.22 – 0.46), 3사분위 AOR = 0.32 (95% CI = 0.22 – 0.47), 4사분위 AOR = 0.28 (95% CI = 0.19 – 0.41)] (Fig. 4; Supplementary Table 3).

여학생에서 디지털 미디어 유형별 사용시간과 운동 부재율은 연관성이 없었다(Fig. 4; Supplementary Table 1-4).

Insert Figure 4 here.

인터넷게임 시간에 대해 거짓보고가 의심되는 대상자 116명을 제외한 민감도 분석에서 남학생은 인터넷게임 비사용군에 비해 인터넷게임 사용시간이 가장 높은 4사분위군에서 중강도 운동률이 유의하게 낮았으며(*p* = 0.038)(Fig. 4A), 인터넷게임 사용시간과 중강도 운동률 사이의 음의 경향성이 있었다(*p* = 0.011)(Fig. 4A). 다중 로지스틱 회귀분석에서도 남학생의 인터넷게임 사용시간 4사분위군이 인터넷게임 비사용군에 비해 운동 부재가 유의하게 높았다[AOR = 1.61 (95% CI = 1.01 – 2.59)](Supplementary table 5).

Insert Figure 5 here.

**고찰**

청소년에서 디지털 미디어 유형별로 디지털 미디어 사용시간에 따라 중강도 신체운동률에 차이가 있는지 파악한 본 연구에서 남학생은 인터넷게임 사용시간에 따라 중강도 운동률에 유의한 차이가 없었지만 게임 스트리밍 시청에서는 게임 스트리밍 과다시청군에서 미시청군에 비해 중강도 운동률이 유의하게 감소하였다. 반대로, 메신저 유형에서는 메신저 비사용군에 비하여 메신저 2,3,4분위 사용군에서 중강도 운동률이 유의하게 증가하였고, 소셜미디어 유형에서는 소셜미디어 비사용군에 비해 모든 사용시간군에서 중강도 운동률이 유의하게 증가하였다.

WHO 청소년 신체활동 가이드라인라인에서는 매일 평균 60분 이상 중등도의 신체활동, 주 3일 이상 격렬한 강도의 유산소 또는 근육 운동을 권장하고 있으며 [20], WHO 기준에 따른 한국 청소년의 신체활동률은 5.8%이다 [2]. 본 연구에서의 결과 변수는 1회 30분 이상 주 2회 이상의 규칙적인 주기로 실시하는 중강도 운동으로, 그 조작적 정의가 WHO 신체활동 가이드라인 기준과 차이가 있다. 본 연구의 결과변수와 거의 동일한 정의를 가지고 본 연구와 동일한 시점에 조사되었던 2016년 국민생활체육조사 자료 [21]에 따르면, 중학생의 규칙적 체육활동 참여율은 44.7%로, 본 연구의 중강도 운동률 45.1%와 유사함을 확인하였다.

자가 보고형 도구에 있어 청소년은 게임 사용과 같이 사회적으로 바람직하지 않아 보이는 위험 행동을 과소 보고 할 가능성이 확인되었고 [19], 특히 게임시간에 대해 거짓말하는 증상은 인터넷게임 사용장애의 진단 기준 중 하나이다. 또한 소셜미디어 사용에 대한 자가보고에서 사용시간를 과대평가하는 경향이 보고되었으나 [42], 게임 스트리밍 시청시간에 대한 거짓보고나 경향성은 현재까지 검토되지 않았다. 다만 게임 동영상을 과도하게 시청하는 행위는 인터넷게임 과다 사용와는 달리 아직은 한국 사회에서 부정적 시각으로 바라보지 않기 때문에 게임 스트리밍 시청시간의 측정에서 거짓보고율은 낮았을 것으로 예상하였다.

인터넷게임 과다 사용자에서 거짓보고에 의한 연관성 왜곡을 확인하기 위하여 거짓보고가 의심되는 인터넷게임 문항에 불일치가 있는 대상자 116명을 제외한 1,721 명을 대상으로 분석한 군에서는 게임 스트리밍 유형과 같이 인터넷게임 과다사용군에서 중강도 운동률이 유의하게 감소하였다.

9세와 15세 아동 및 청소년의 전국 대표 표본 3,920명을 대상으로, 자가보고로 TV, PC와 게임을 비롯한 하루 총 스크린 시간과 가속도계를 통해 측정된 중강도 신체활동과의 연관성을 알아본 노르웨이 단면연구에서는 하루 총 스크린 시간이 1시간 증가함에 따라 하루 평균 2분씩 신체활동이 감소하는 음의 연관성을 보고하였다 [23]. 본 연구의 민감도 분석에서 인터넷게임 사용시간과 중강도 신체운동률 사이 음의 연관성을 확인하였고, 비사용자에 비하여 과다사용자에서 중강도 신체운동이 현저하게 감소하였다.

반대로 남학생에서 메신저 및 소셜미디어 사용시간이 증감함에 따라 중강도 운동률이 증가하였다. 홍콩의 중학생 및 대학생 187명을 대상으로 스마트폰의 미디어별 노출시간과 가속도계로 측정한 중강도 신체활동 시간의 연관성을 알아본 단면연구 [11]에서는 메신저 및 소셜 미디어 사용시간의 증가와 중강도 신체활동의 증가가 연관성이 있는 것으로 나타나 본 연구결과와 일치하였다. 소셜 미디어를 사용하는 영국 청소년에 대한 질적연구에서는 남학생의 소셜미디어 사용동기가 친구와의 의사소통, 그리고 외모와 관련된 건강정보에 대한 접근임을 보고하였다 [24]. 신체활동이 많은 사람들은 사진 및 건강정보를 공유하기 위해 메신저 및 소셜미디어를 많이 사용할 수 있기 때문에 [7] 소셜미디어 사용과 중강도 신체활동 사이에 양의 연관성이 나타난 것일 수도 있다.

여학생에서는 인터넷게임, 메신져, 소셜미디어, 게임 스트리밍 시청 모두 미디어 사용여부와 사용시간에 따른 중강도 신체운동률 사이의 유의한 연관성이 없었다. 중고등학생 19,543명을 분석한 미국 연구에서는 신체활동이 활발한 학생들에서는 소셜미디어 사용빈도가 높을수록 신체활동이 증가되고, 신체활동이 거의 없는 학생들에서는 소셜미디어 사용빈도가 높을수록 신체활동이 감소되는 결과를 보임으로서 신체활동 수준에 따라 차이가 있음을 보고하였다 [25]. 북미와 유럽 39개국의 11-15세 청소년 200,615명의 자료를 분석한 노르웨이 연구에서는 남녀 모두에서 TV, PC, 게임을 사용한 총 시간이 2시간을 초과하면 중강도 신체활동이 감소함을 보고하였다 [26]. WHO 신체활동 가이드라인 수준을 충족하는 한국 여학생의 비율은 겨우 2.8%로, 북미 대표국가인 미국 여학생의 19.5%와 남유럽 대표국가인 이탈리아 여학생의 8.5%보다도 현저히 낮은 수준이다 [2]. 따라서 한국 청소년 여학생에서 신체활동이 전반적으로 부족하기 때문에 디지털 미디어 유형에 따른 중강도 운동률과의 연관성을 관찰할 수 없었다.

남학생 인터넷게임 비사용군의 중강도 신체운동 부재율이 35.1%였다. 따라서 연구에서 도출된 연관성의 크기인 오즈비 값은 상대위험도를 과대 추정할 수 있다 [27]. 오즈비(OR)가 1.62 일때 환산한 상대위험도(RR)는 1.33 이었다. 남자 중학생에서 인터넷게임 과다사용이 중강도 신체운동 부재의 직접 원인이라고 가정할 때 인터넷게임 과다사용은 운동 부재율을 1.33배 증가시키는 것으로 나타났다.

이 연구의 강점은 미디어 유형에 대한 구분이 없는 총 스크린 시간 대신 연구대상자가 선호하는 디지털 미디어 유형별 사용시간을 평가하여 디지털 미디어 유형별로 중강도 운동률과의 연관성을 파악한 것이다. 둘째, 기존 연구에서는 신체활동에 대한 정의가 모호하고 광범위하였다. 그러나 본 연구에서는 중학생에서 디지털 미디어 사용시간과의 연관성을 파악하기 위해 중강도 신체운동으로 한정하였다.

반면 이 연구의 한계점으로는 첫째, 본 연구는 단면연구로서 인과관계가 아닌 연관성만을 확인할 수 있었다. 둘째, 디지털 미디어 사용시간과 중강도 운동참여에 대해 주관적으로 측정하였기 때문에, 학생들이 사회적 인정 욕구로 인하여 운동참여를 부풀리거나 사회적 바람직함에 대한 바이어스로 미디어 사용시간을 축소 보고했을 우려가 있다. 이에 인터넷게임 사용시간에 대한 거짓보고를 제외하기 위해 민감도 분석을 실시하여 연관성을 파악하였다. 셋째, 노출변수인 미디어 유형에 대한 독립적 효과를 파악하는데 있어 다른 유형의 미디어 사용시간을 보정하지 않았다. 인터넷 게임과 게임 스트리밍 시청, 메신저와 소셜미디어와 같이 2개 이상의 미디어 사용이 하나의 행위처럼 연계되어 있기 때문에 분리하여 분석하는 것은 현실적으로 어려움이 있었다. 이에 따라 관찰된 미디어 사용 형태를 오즈비로 산출하고자 하였다. 넷째, 디지털 미디어 사용 패턴과 중강도 신체활동은 국가, 지역, 개인적 특성에 영향을 받을 수 있기 때문에 한국 수도권 지역의 학교 재학 중인 학생들을 대상으로 한 이 연구를 다른 국가, 시골 지역, 학교 밖 청소년에 대해 결과를 일반화할 때 주의가 필요하다. 다섯째, 여학생에서의 소셜 미디어 사용과 중강도 미만의 신체활동 사이의 연관성을 분석하지 못했다. 소셜미디어에 대한 여학생의 관심과 평균 사용량이 높기 때문에 더 낮은 강도의 신체활동과의 연관성에 대한 좋사가 필요하다. 본 연구에서는 신체활동 수준에 대해 측정하지 못해서 그 부분을 확인할 수 없었다. 향후에는 성별 및 신체활동 수준에 따른 소셜미디어 사용과의 관계에 대한 연구가 필요하리라 생각한다.

남자 중학생에서 온라인 게임사용이 과다하고, 게임 스트리밍을 과다 시청할 때는 중강도 운동률이 감소할 가능성이 있다. 이에 대한 향후 연구와 디지털 미디어 사용 가이드라인 제정이 필요하다.

**Conflicts of Interest**

The authors have no conflicts of interest to declare for this study

**Author Contributions**

Conceptualization: GK, HWY

Data curation: GK. HWY, HJ

Formal analysis: GK, HWY, HJ

Funding acquisition: HWY

Methodology: GK, HWY, HJ

Project administration: HWY

Visualization: GK, HWY

Writing – original draft: GK

Writing – review & editing: GK, HWY, HJ

**ORCID**

Gyeongmin Kim https://orcid.org/0000-0003-2944-8063

Hyunsuk Jeong https://orcid.org/0000-0001-5274-3816

Hyeon Woo Yim https://orcid.org/0000-0002-3646-8161

**참고문헌**

1. 2018 Physical Activity Guidelines Advisory Committee. "2018 physical activity guidelines advisory committee scientific report." (2018): F2-F33.

2.Guthold R, Stevens GA, Riley LM, Bull FC. Global trends in insufficient physical activity among adolescents: a pooled analysis of 298 population-based surveys with 1·6 million participants. Lancet Child Adolesc Health 2020;4:23-35.

3.van Sluijs EM, Ekelund U, Crochemore-Silva I, Guthold R, Ha A, Lubans D, et al. Physical activity behaviours in adolescence: current evidence and opportunities for intervention. The Lancet 2021;398:429-442.

4.Rachmi CN, Agho KE, Li M, Baur LA. Stunting, underweight and overweight in children aged 2.0–4.9 years in Indonesia: prevalence trends and associated risk factors. PloS one 2016;11:e0154756.

5.Dalene KE, Anderssen SA, Andersen LB, Steene-Johannessen J, Ekelund U, Hansen BH, et al. Cross-sectional and prospective associations between sleep, screen time, active school travel, sports/exercise participation and physical activity in children and adolescents. BMC public health 2018;18:1-10.

6.Dahlgren A, Sjöblom L, Eke H, Bonn SE, Trolle Lagerros Y. Screen time and physical activity in children and adolescents aged 10–15 years. PloS one 2021;16:e0254255.

7.Lee PH, Tse AC, Wu CS, Mak YW, Lee U. Temporal association between objectively measured smartphone usage, sleep quality and physical activity among Chinese adolescents and young adults. Journal of Sleep Research 2021;30:e13213.

8.Serrano-Sanchez JA, Martí-Trujillo S, Lera-Navarro A, Dorado-García C, González-Henríquez JJ, Sanchís-Moysi J. Associations between screen time and physical activity among Spanish adolescents. PloS one 2011;6:e24453.

9.Spengler S, Mess F, Woll A. Do media use and physical activity compete in adolescents? Results of the MoMo study. PLoS One 2015;10:e0142544.

10.Xie H, Scott JL, Caldwell LL. Urban adolescents’ physical activity experience, physical activity levels, and use of screen-based media during leisure time: A structural model. Frontiers in psychology 2018;8:2317.

11.Twenge JM, Martin GN. Gender differences in associations between digital media use and psychological well-being: Evidence from three large datasets. J Adolesc 2020;79:91-102.

12.Jeong H, Yim HW, Jo SJ, Lee SY, Kim E, Son HJ, et al. Study protocol of the internet user Cohort for Unbiased Recognition of gaming disorder in Early adolescence (iCURE), Korea, 2015-2019. BMJ Open 2017;7:e018350.

13.Ministry of Science, ICT and Future planning, Korea internet security agency. 2017 Survey on the Internet Usage. Sejong; 2017. 56-66p. Report No. 120005.

14. Kim Y, Yang Y, Park H, Kim J. Development of physical activity guidelines and self-prescription guides for Korean. Seoul: Korea Health Promotion Institute 2012

15.Cho S, Lee Y. Development of the Korean form of the Kovacs' Children's Depression Inventory. J Korean Neuropsychiatr Assoc 1990;29:943-956.

16.Cho S. CJ Development of State-Trait Anxiety Scale for Korean Children. J Med Seoul Natinal University 1989;14

17.Seo S, Kwon S. Validation study of the Korean version of the aggression questionnaire. Korean J Clin Psychol 2002;21:487-501.

18.Van Den Eijnden R, Koning I, Doornwaard S, Van Gurp F, Ter Bogt T. The impact of heavy and disordered use of games and social media on adolescents’ psychological, social, and school functioning. Journal of behavioral addictions 2018;7:697-706.

19.Jeong H, Yim HW, Lee S-Y, Lee HK, Potenza MN, Kwon J-H, et al. Discordance between self-report and clinical diagnosis of Internet gaming disorder in adolescents. Scientific Reports 2018;8:1-8.

20.Organization WH. Physical activity [cited 2022 15 Nov]. Available from: https://www.who.int/news-room/fact-sheets/detail/physical-activity.

21.2016 National sport participation survey in Korea.: Ministry of Culture, Sports and Tourism (KR); 2016 Dec. Report No : 11-1371000-000289-11

22.Katapally TR, Chu LM. Methodology to derive objective screen-state from smartphones: a SMART platform study. International journal of environmental research and public health 2019;16:2275.

23.Hansen BH, Kolle E, Steene-Johannessen J, Dalene KE, Ekelund U, Anderssen SA. Monitoring population levels of physical activity and sedentary time in Norway across the lifespan. Scand J Med Sci Sports 2019;29:105-112.

24.Goodyear V, Quennerstedt M. #Gymlad - young boys learning processes and health-related social media. Qual Res Sport Exerc Health 2020;12:18-33.

25.Shimoga SV, Erlyana E, Rebello V. Associations of Social Media Use With Physical Activity and Sleep Adequacy Among Adolescents: Cross-Sectional Survey. J Med Internet Res 2019;21:e14290.

26.Melkevik O, Torsheim T, Iannotti RJ, Wold B. Is spending time in screen-based sedentary behaviors associated with less physical activity: a cross national investigation. International Journal of Behavioral Nutrition and Physical Activity 2010;7:1-10.

27.Grimes DA, Schulz KF. Making sense of odds and odds ratios. Obstet Gynecol 2008;111:423-426.
