# Supplementary material for: Associations between digital media use and lack of physical exercise among middle-school adolescents in Korea
Source: Epidemiol Health. 2023 Jan 10;45:e2023012. doi: 10.4178/epih.e2023012 (PMC10581895; doi:10.4178/epih.e2023012)
Supplement: Supplementary Material 1. — Associations between time spent on playing Internet game and lack of moderate intensity physical exercise (n=1,837) [file epih-45-e2023012-Supplementary-1.docx]

**SUPPLEMENTARY MATERIALS**

**Supplementary Material 1. Associations between time spent on playing Internet game and lack of moderate intensity physical exercise (n=1,837)**

| **Internet** |  |  |  |  |  |
| --- | --- | --- | --- | --- | --- |
| **game** | Criteria | n (%) | Lack of exercise^a^ | Crude | Adjusted ^c^ |
|  | (min) |  | n(%) ^b^ | OR (95% CI) | aOR (95% CI) |
| Boys | None | 130 (12.3) | 49 (37.7) | 1 | 1 |
| (n=1,055) | 0 to < 60 | 179 (17.0) | 65 (36.3) | 0.94 (0.59-1.50) | 0.97 (0.61-1.56) |
|  | 60 to < 90 | 236 (22.4) | 93 (39.4) | 1.08 (0.69-1.67) | 1.08 (0.69-1.69) |
|  | 90 to < 150 | 266 (25.2) | 111 (41.7) | 1.18 (0.77-1.82) | 1.17 (0.76-1.80) |
|  | ≥ 150 | 244 (23.1) | 114 (46.7) | 1.45 (0.94-2.24) | 1.43 (0.92-2.24) |
| Girls | None | 331 (42.3) | 234 (70.7) | 1 | 1 |
| (n=782) | 0 to < 30 | 107 (13.7) | 79 (73.8) | 1.17 (0.72-1.91) | 1.18 (0.72-1.93) |
|  | 30 to < 60 | 93 (11.9) | 72 (77.4) | 1.42 (0.83-2.44) | 1.38 (0.80-2.38) |
|  | 60 to < 120 | 128 (16.4) | 97 (75.8) | 1.30 (0.81-2.07) | 1.32 (0.82-2.12) |
|  | ≥ 120 | 123 (15.7) | 94 (76.4) | 1.34 (0.83-2.17) | 1.37 (0.84-2.24) |

* : *p* < .05 ** : *p* < .01 *** : *p* < .001

a < participating in moderate intensity physical exercise on 2 days of the week (more than 30 minutes at a time)

b n (%) for lack of exercise within the level of time spent on media

c Adjusted for maternal educational level, aggression(AQ), children's depression(CDI), state anxiety(SAIC), and time spent on private tutoring.
