# Supplementary material for: Associations between digital media use and lack of physical exercise among middle-school adolescents in Korea
Source: Epidemiol Health. 2023 Jan 10;45:e2023012. doi: 10.4178/epih.e2023012 (PMC10581895; doi:10.4178/epih.e2023012)
Supplement: Supplementary Material 2 — Associations between time spent on Messenger and lack of moderate intensity physical exercise (n=1,837) [file epih-45-e2023012-Supplementary-2.docx]

**Supplementary Material 2. Associations between time spent on Messenger and lack of moderate intensity physical exercise (n=1,837)**

| **Messenger** |  |  |  |  |  |
| --- | --- | --- | --- | --- | --- |
|  | Criteria | n (%) | Lack of exercise ^a^ | Crude | Adjusted ^c^ |
|  | (min) |  | n(%) ^b^ | OR (95% CI) | aOR (95% CI) |
| Boys | None | 136 (12.9) | 80 (58.8) | 1 | 1 |
| (n=1,055) | 0 to < 10 | 134 (12.7) | 67 (50.0) | 0.70 (0.43-1.13) | 0.73 (0.45-1.19) |
|  | 10 to < 30 | 256 (24.3) | 103 (40.2) | 0.47 (0.31-0.72) ^***^ | 0.50 (0.33-0.77) ^**^ |
|  | 30 to < 60 | 190 (18.0) | 62 (32.6) | 0.34 (0.22-0.54) ^***^ | 0.36 (0.23-0.57) ^***^ |
|  | ≥ 60 | 339 (32.1) | 120 (35.4) | 0.38 (0.25-0.58) ^***^ | 0.40 (0.27-0.61) ^***^ |
| Girls | None | 48 (6.1) | 34 (70.8) | 1 | 1 |
| (n=782) | 0 to < 30 | 176 (22.5) | 130 (73.9) | 1.16 (0.57-2.36) | 1.16 (0.57-2.36) |
|  | 30 to < 60 | 154 (19.7) | 114 (74.0) | 1.17 (0.57-2.41) | 1.15 (0.56-2.37) |
|  | 60 to < 120 | 186 (23.8) | 139 (74.7) | 1.22 (0.60-2.46) | 1.21 (0.59-2.46) |
|  | ≥ 120 | 218 (27.9) | 159 (72.9) | 1.11 (0.56-2.21) | 1.12 (0.56-2.26) |

* : *p* < .05 ** : *p* < .01 *** : *p* < .001

a < participating in moderate intensity physical exercise on 2 days of the week (more than 30 minutes at a time)

b n (%) for lack of exercise within the level of time spent on media

c Adjusted for maternal educational level, aggression(AQ), children's depression(CDI), state anxiety(SAIC), and time spent on private tutoring.
