# Supplementary material for: Associations between digital media use and lack of physical exercise among middle-school adolescents in Korea
Source: Epidemiol Health. 2023 Jan 10;45:e2023012. doi: 10.4178/epih.e2023012 (PMC10581895; doi:10.4178/epih.e2023012)
Supplement: Supplementary Material 3 — Associations between time spent on Social media and lack of moderate intensity physical exercise (n=1,837) [file epih-45-e2023012-Supplementary-3.docx]

**Supplementary Material 3. Associations between time spent on Social media and lack of moderate intensity physical exercise (n=1,837)**

| **Social media** |  |  |  |  |  |
| --- | --- | --- | --- | --- | --- |
|  | Criteria | n (%) | Lack of exercise ^a^ | Crude | Adjusted ^c^ |
|  | (min) |  | n(%) ^b^ | OR (95% CI) | aOR (95% CI) |
| Boys | None | 306 (29.0) | 186 (60.8) | 1 | 1 |
| (n=1,039) | 0 to < 20 | 159 (15.1) | 63 (39.6) | 0.42 (0.28-0.63) ^***^ | 0.44 (0.30-0.65) ^***^ |
|  | 20 to < 60 | 207 (19.6) | 65 (31.4) | 0.30 (0.20-0.43) ^***^ | 0.32 (0.22-0.46) ^***^* |
|  | 60 to < 90 | 189 (17.9) | 61 (32.3) | 0.31 (0.21-0.45) ^***^ | 0.32 (0.22-0.47) ^***^ |
|  | ≥ 90 | 194 (18.4) | 57 (29.4) | 0.27 (0.18-0.40) ^***^ | 0.28 (0.19-0.41) ^***^ |
| Girls | None | 100 (12.8) | 72 (72.0) | 1 | 1 |
| (n=682) | 0 to < 35 | 168 (21.5) | 117 (69.6) | 0.89 (0.52-1.54) | 0.87 (0.50-1.51) |
|  | 35 to < 90 | 168 (21.5) | 127 (75.6) | 1.21 (0.69-2.11) | 1.19 (0.68-2.09) |
|  | 90 to < 180 | 147 (18.8) | 118 (80.3) | 1.58 (0.87-2.87) | 1.51 (0.83-2.76) |
|  | ≥ 180 | 199 (25.5) | 142 (71.4) | 0.97 (0.57-1.65) | 0.96 (0.56-1.65) |

* : *p* < .05 ** : *p* < .01 *** : *p* < .001

a < participating in moderate intensity physical exercise on 2 days of the week (more than 30 minutes at a time)

b n (%) for lack of exercise within the level of time spent on media

c Adjusted for maternal educational level, aggression(AQ), children's depression(CDI), state anxiety(SAIC), and time spent on private tutoring.
