# Supplementary material for: Associations between digital media use and lack of physical exercise among middle-school adolescents in Korea
Source: Epidemiol Health. 2023 Jan 10;45:e2023012. doi: 10.4178/epih.e2023012 (PMC10581895; doi:10.4178/epih.e2023012)
Supplement: Supplementary Material 4 — Associations between time spent watching Game streaming and lack of moderate intensity physical exercise (n=1,837) [file epih-45-e2023012-Supplementary-4.docx]

**Supplementary Material 4. Associations between time spent watching Game streaming and lack of moderate intensity physical exercise (n=1,837)**

| **Game streaming** |  |  |  |  |  |
| --- | --- | --- | --- | --- | --- |
|  | Criteria | n (%) | Lack of exercise ^a^ | Crude | Adjusted ^c^ |
|  | (min) |  | n(%) ^b^ | OR (95% CI) | aOR (95% CI) |
| Boys | None | 272 (25.8) | 105 (39.0) | 1 | 1 |
| (n=1,055) | 0 to < 30 | 148 (14.0) | 57 (38.5) | 0.98 (0.65-1.48) | 1.03 (0.68-1.56) |
|  | 30 to < 60 | 174 (16.5) | 65 (37.4) | 0.93 (0.63-1.38) | 0.95 (0.64-1.41) |
|  | 60 to < 120 | 251 (23.8) | 92 (36.7) | 0.91 (0.64-1.29) | 0.93 (0.65-1.33) |
|  | ≥ 120 | 210 (19.9) | 112 (53.3) | 1.79 (1.24-2.58) ^**^ | 1.83 (1.26-2.65) ^**^ |
| Girls | None | 548 (70.1) | 402 (73.4) | 1 | 1 |
| (n=782) | 0 to < 30 | 41 (5.2) | 31 (75.6) | 1.13 (0.54-2.35) | 1.18 (0.56-2.48) |
|  | 30 to < 60 | 50 (6.4) | 42 (84.0) | 1.91 (0.88-4.16) | 1.85 (0.84-4.05) |
|  | 60 to < 120 | 74 (9.5) | 50 (67.6) | 0.76 (0.45-1.28) | 0.80 (0.47-1.37) |
|  | ≥ 120 | 69 (8.8) | 51 (73.9) | 1.03 (0.58-1.82) | 1.01 (0.56-1.81) |

* : *p* < .05 ** : *p* < .01 *** : *p* < .001

a < participating in moderate intensity physical exercise on 2 days of the week (more than 30 minutes at a time)

b n (%) for lack of exercise within the level of time spent on media

c Adjusted for maternal educational level, aggression(AQ), children's depression(CDI), state anxiety(SAIC), and time spent on private tutoring.
