# Supplementary material for: Associations between digital media use and lack of physical exercise among middle-school adolescents in Korea
Source: Epidemiol Health. 2023 Jan 10;45:e2023012. doi: 10.4178/epih.e2023012 (PMC10581895; doi:10.4178/epih.e2023012)
Supplement: Supplementary Material 5 — Sensitivity analysis of associations between time spent on playing Internet game and lack of moderate intensity physical exercise (n=1,721) [file epih-45-e2023012-Supplementary-5.docx]

**Supplementary Material 5. Sensitivity analysis of associations between time spent on playing Internet game and lack of moderate intensity physical exercise (n=1,721)**

| **Internet** |  |  |  |  |  |
| --- | --- | --- | --- | --- | --- |
| **game** | Criteria | n (%) | Lack of exercise ^a^ | Crude | Adjusted ^c^ |
|  | (min) |  | n(%) ^b^ | OR (95% CI) | aOR (95% CI) |
| Boys | None | 114 (11.0) | 40 (35.1) | 1 | 1 |
| (n=1,039) | 0 to < 60 | 179 (17.2) | 65 (36.3) | 1.06 (0.65-1.72) | 1.10 (0.67-1.81) |
|  | 60 to < 90 | 236 (22.7) | 93 (39.4) | 1.20 (0.76-1.92) | 1.21 (0.76-1.94) |
|  | 90 to < 150 | 266 (25.6) | 111 (41.7) | 1.33 (0.84-2.09) | 1.29 (0.81-2.05) |
|  | ≥ 150 | 244 (23.5) | 114 (46.7) | 1.62 (1.03-2.57) ^*^ | 1.61 (1.01-2.59) ^*^ |
| Girls | None | 231 (33.9) | 162 (70.1) | 1 | 1 |
| (n=682) | 0 to < 30 | 107 (15.7) | 79 (73.8) | 1.20 (0.72-2.01) | 1.21 (0.72-2.04) |
|  | 30 to < 60 | 93 (13.6) | 72 (77.4) | 1.46 (0.83-2.56) | 1.41 (0.80-2.48) |
|  | 60 to < 120 | 128 (18.8) | 97 (75.8) | 1.33 (0.81-2.18) | 1.31 (0.79-2.16) |
|  | ≥ 120 | 123 (18.0) | 94 (76.4) | 1.38 (0.84-2.28) | 1.36 (0.81-2.29) |

* : *p* < .05 ** : *p* < .01 *** : *p* < .001

a < participating in moderate intensity physical exercise on 2 days of the week (more than 30 minutes at a time)

b n (%) for lack of exercise within the level of time spent on media

c Adjusted for maternal educational level, aggression(AQ), children's depression(CDI), state anxiety(SAIC), and time spent on private tutoring.
